# Individual Differences in Working Memory and the N2pc

**DOI:** 10.3389/fnhum.2021.620413

**Published:** 2021-03-11

**Authors:** Jane W. Couperus, Kirsten O. Lydic, Juniper E. Hollis, Jessica L. Roy, Amy R. Lowe, Cindy M. Bukach, Catherine L. Reed

**Affiliations:** ^1^Mt. Holyoke College, South Hadley, MA, United States; ^2^Hampshire College, Amherst, MA, United States; ^3^Psychology Department, University of Richmond, Richmond, VA, United States; ^4^Claremont McKenna College, Claremont, CA, United States

**Keywords:** event related potentials, visual working memory, spatial working memory, visual search, N2pc

## Abstract

The lateralized ERP N2pc component has been shown to be an effective marker of attentional object selection when elicited in a visual search task, specifically reflecting the selection of a target item among distractors. Moreover, when targets are known in advance, the visual search process is guided by representations of target features held in working memory at the time of search, thus guiding attention to objects with target-matching features. Previous studies have shown that manipulating working memory availability via concurrent tasks or within task manipulations influences visual search performance and the N2pc. Other studies have indicated that visual (non-spatial) vs. spatial working memory manipulations have differential contributions to visual search. To investigate this the current study assesses participants' visual and spatial working memory ability independent of the visual search task to determine whether such individual differences in working memory affect task performance and the N2pc. Participants (*n* = 205) completed a visual search task to elicit the N2pc and separate visual working memory (VWM) and spatial working memory (SPWM) assessments. Greater SPWM, but not VWM, ability is correlated with and predicts higher visual search accuracy and greater N2pc amplitudes. Neither VWM nor SPWM was related to N2pc latency. These results provide additional support to prior behavioral and neural visual search findings that spatial WM availability, whether as an ability of the participant's processing system or based on task demands, plays an important role in efficient visual search.

## Introduction

Every day we are presented with visual scenes that challenge our attentional system, whether that is finding our cell phone on a cluttered desk or searching for a friend in a crowd. To function, we need an attentional system that can quickly and accurately assess the visual field to determine what in the environment is currently relevant. Selecting out relevant information during a visual search requires identifying objects as well as resolving conflicts between competing objects using selective attention and working memory. In the context of visual search, selective attention has often been examined through the N2 posterior contralateral component, or the N2pc. The N2pc has been shown to be an effective electrophysiological marker of attentional object selection when presented with a visual search task, reflecting selection of a target item among distractors (Luck and Hillyard, 1994a,b; Eimer et al., 2011).

A number of theories suggest that working memory availability and attention, both limited capacity systems, work together to allow for efficient visual search (Oh and Kim, 2004; Henare et al., 2018). Models of visual search propose that a when a target is known in advance, the visual search process is guided by representations of target features held in working memory at the time of search, thus guiding attention to objects with target-matching features (Grubert and Eimer, 2016). In other words, the search target template is held in working memory which directs or biases perceptual mechanisms to locate and process items possessing the target's features in the search array, compare the working memory template to potential candidates, and categorize them (Bundesen, 1990; Desimone and Duncan, 1995; Woodman and Luck, 2004; Luria and Vogel, 2011). Animal physiological research has shown that working memory influences the allocation of selective attention (Chelazzi et al., 1998; Desimone, 1998) and human behavioral work has shown that selective attention is shifted toward an object held in working memory (Downing, 2000). Thus, the greater availability of working memory resources allows for increased ability to select targets while suppressing and disengaging from distractors (Carrasco, 2011). Additional support for the impact of working memory on visual search comes from evidence that increasing load on a concurrent working memory task will lead to decreased performance on the search task and more interference from irrelevant distractors (de Fockert et al., 2001; Stins et al., 2004; Ahmed and de Fockert, 2012).

However, not all human studies find that manipulations of working memory load impairs search efficiency (Woodman et al., 2001). Interactions between visual search and working memory may depend on which aspect of working memory is manipulated, as well as the experimental task. When studying working memory in visual search tasks, most studies manipulate the load on visual working memory in which visual properties of items are held in working memory but not their locations. However, also relevant to visual search is spatial working memory where the locations of visually presented items are held in working memory but not their visual features. Woodman et al. (2001) found no influence of working memory on visual search performance when the visual working memory task used a visual change detection task that had no spatial components (i.e., visual working memory). In contrast, Oh and Kim (2004) combined visual search and working memory tasks, comparing the effects of spatial and visual (non-spatial) working memory load. A memory array was presented, followed by a search array where participants searched for a target figure, followed by a memory probe. For the spatial working memory task, participants memorized the locations of four items in four of eight possible locations and reported if the items in the test probe matched the locations of the items in the memory array. For the visual memory task, participants memorized the color of four items presented in four central locations and reported if the items in the test probe had the same colors. Increased spatial working memory load impaired both the behavioral search process and spatial working memory accuracy, but visual working memory load did not, suggesting that spatial working memory can produce distinct effects from visual working memory. Working memory used during visual search and general spatial working memory storage may rely either on common limited-capacity mechanisms or from a common demand for spatial attention (Awh et al., 1998).

Working memory has long been theorized to be a multicomponent system (Engle, 2002; Cowan, 2008). Both behavioral and neuropsychological studies have further supported separable subsystems for storing both behavioral and neuropsychological non-spatial and spatial visual representations (Farah et al., 1988; Baddeley and Logie, 1999; Carlesimo et al., 2001). To show functionally separate contributions of spatial vs. visual working memory to a task, researchers have often employed dual-task interference paradigms (e.g., Logie and Marchetti, 1991). For example, Woodman and Luck (2004) conducted a dual-task behavioral study in which participants performed either a spatial change detection task or a visual change detection task while performing visual search. No interference between tasks was found for holding color or shape representations in memory during visual search. In contrast, holding spatial locations in memory interfered with visual search efficiency and increasing the number of items in the visual search array decreased spatial task accuracy. This interference suggests that both visual search and spatial change detection tasks required access to a common limited capacity process. They propose that spatial locations are held in working memory by visuospatial attention and these locations may be important for directing attention in the search task.

Researchers have also turned to electrophysiology to provide a potentially more sensitive measure of how specific aspects of working memory may be related to selective attention in the context of visual search. Studies suggest the N2pc event-related potential (ERP) component reflects attentional-filtering operations as a target is selected among distractors (Luck and Hillyard, 1994a,b; Eimer, 1996; Luck et al., 1997; Hopf et al., 2002; Boehler et al., 2011). The N2pc is recorded over lateral occipital scalp regions when a search display appears and is seen ~175–300 ms after the onset of the display. The selection of the attended item is reflected in a component described as a greater negative activity contralateral as compared to ipsilateral to the attended item. It is generally agreed that the N2pc reflects target selection, but it may also reflect the relative contributions of selective target enhancement and suppression of distractors involved in visual search (Eimer, 1996; Hickey et al., 2009; Henare et al., 2018; Li et al., 2018). Thus, examination of the N2pc in addition to behavioral performance may provide insight into what specific aspects of working memory may contribute to visual search.

Many studies that examined working memory processes during visual search using the N2pc have used concurrent visual search and visual working memory tasks to manipulate working memory availability. If working memory mechanisms play a role in selective attention during visual search as indicated by the N2pc, then reducing working memory availability should influence both visual search performance and the N2pc, especially in terms of amplitude. Although some studies have shown an impact of visual working memory manipulations on visual search as measured by N2pc amplitudes (e.g., Kuo et al., 2009; Dell'Acqua et al., 2010; Eimer and Kiss, 2010; Shimi et al., 2015; Grubert and Eimer, 2016; Feldmann-Wüstefeld and Vogel, 2019; Salahub et al., 2019), other studies find effects only on other lateralized components (e.g., Emrich et al., 2009; Luria and Vogel, 2011).

In addition, studies have found that working memory load during visual search tasks influenced N2pc latencies (Berggren and Eimer, 2018, 2019, Kumar et al., 2009). For example, Berggren and Eimer (2018, 2019) had participants memorize one or four shapes before either testing their memory or performing a visual search task. One study (2018) asked participants to identify targets by color and the other study (2019) asked participants to discriminate orientation of a rectangular bar defined by location. For both studies, N2pc latency was delayed in high load conditions, suggesting that load interfered with the activation of search templates. Although this research did not examine individual differences it implies that the ability to handle load may depend on individual differences in both visual and spatial working memory ability.

A correlation between measures on two independent tasks can also provide compelling evidence for common mechanisms. This individual differences approach has been applied to investigate how participant working memory affects visual search. In these studies, working memory ability was assessed independently of the visual search task. Those with low working memory capacity should have less overall available working memory, thereby leading to reduced performance and/or greater condition interference (Kane and Engle, 2003; de Fockert, 2013; Shipstead et al., 2016). For visual search tasks, low working memory availability delays the deployment of attention to relevant targets (Heitz and Engle, 2007; Scalf et al., 2011) and may increase the attentional capture by distractors (Fukuda and Vogel, 2011).

Although individual differences in working memory capacity may be related to N2pc amplitude when working memory is taxed during visual search (Shimi et al., 2015; Heuer and Schubö, 2016), only a few studies have assessed visual working memory capacity independently from the visual search task and examined the relation to lateralized ERP components. Luria and Vogel (2011) found a strong relationship between visual working memory capacity and CDA amplitudes (contralateral delay activity associated with the number of representations in visual working memory) during low, medium, and high difficulty visual search tasks. Importantly, this relation was found when visual working memory was assessed behaviorally via a visual change detection task independently of the search. However, the N2pc measured during visual search did not correlate with individual differences in visual working memory capacity. Another study by Gaspar et al. (2016) examined how individual differences in visual working memory capacity influenced lateralized ERP components. They, like Luria and Vogel (2011) assessed visual working memory capacity via a visual change detection task conducted independently from the EEG/ERP visual search task. Although they also did not show a relationship between visual working memory capacity and the N2pc, they did show a correlation with Pd amplitudes (a positivity related to distractor suppression; Hickey et al., 2009) such that those with higher visual working memory scores also showed a greater Pd response. However, both of these individual difference studies examined visual working memory capacity without a spatial component. As a result, the relationship between working memory capacity and the N2pc remains unclear because visual search may be guided by both visual and spatial working memory.

Thus, the role of visual and spatial working memory in visual search and how they moderate the N2pc remains in question. Although prior studies manipulating task demands within the visual search task itself demonstrate memory load affects visual search and the N2pc, we are unaware of any EEG/ERP studies that have directly examined whether individual differences in visual vs. spatial working memory ability differentially relate to visual search performance and N2pc amplitude and latency. The purpose of this study was to differentiate the relative contributions of visual working memory (VWM) and spatial working memory (SPWM) on visual search performance and N2pc amplitudes and latencies. We measured working memory via individual differences in working memory ability in the participants and separately measured the N2pc in an EEG/ERP visual search task. We used a large sample to obtain reliable measures of individual differences in both visual working memory and spatial working memory. We predict that greater working memory accuracy overall should lead to increased visual task performance and greater N2pc amplitudes. However, based on previous behavioral studies and the importance of maintaining spatial locations to locate targets, both spatial working memory ability and visual working memory ability may be predictive of N2pc amplitude.

## Methods

### Participants

Event related potential and behavioral data from 242 adults who participated in all aspects of this study as part of a larger research project (for more information on this project see http://pursueerp.com/). Participants were recruited from Hampshire College, University of Richmond, and Claremont McKenna College. All adult participants provided consent prior to participation. All consents and research procedures were approved by the Institutional Review Boards of the participating campuses. All participants were financially compensated for their time.

Prior to statistical analyses, participants were excluded if there were <50% of trials remaining after artifact rejection and correction (5 participants, 2% of total data) or significant noise remained in the data following artifact correction (i.e., the signal to noise ratio was high due to poor recording quality) (27 participants, 11.1% of total data). An additional 5 participants were excluded due to low behavioral performance (i.e., accuracy under 50%). In all, 37 of 242 participants were excluded, leaving 205 participants to be included in analyses (total data loss of 15.3%).

These 205 participants had a mean age of 19.59 (SD = 1.57, range 18–30 yrs) and included 56 males, 117 females, 15 individuals who identified as Non-binary or other, and 17 who did not report gender. Of these participants, 102 self-identified as White, 9 Black, 26 Asian, 17 Hispanic, 4 Other, and 23 who indicated more than one designation; 24 participants did not report race/ethnicity (by choosing the prefer not to say option or leaving the question blank).

### Procedure

Participants completed testing across two testing sessions. In session 1, primary demographic data was collected including age, gender, handedness, vision, and education as well as other self-report measures. In session 2, the visual search task was performed while EEG was collected. Computer-based, validated measures of visual and spatial working memory were performed separately on an IPad without EEG recording (brainbaseline.com; Lee et al., 2012). Participants held the IPad at a comfortable distance on a table in front of them, approximately 50 cm away. For each task, the experimenter made sure the participant understood the instructions and then left the room while the task was performed. Task order was counterbalanced across participants.

### Tasks

#### Visual Search Task

**Figure 1:**
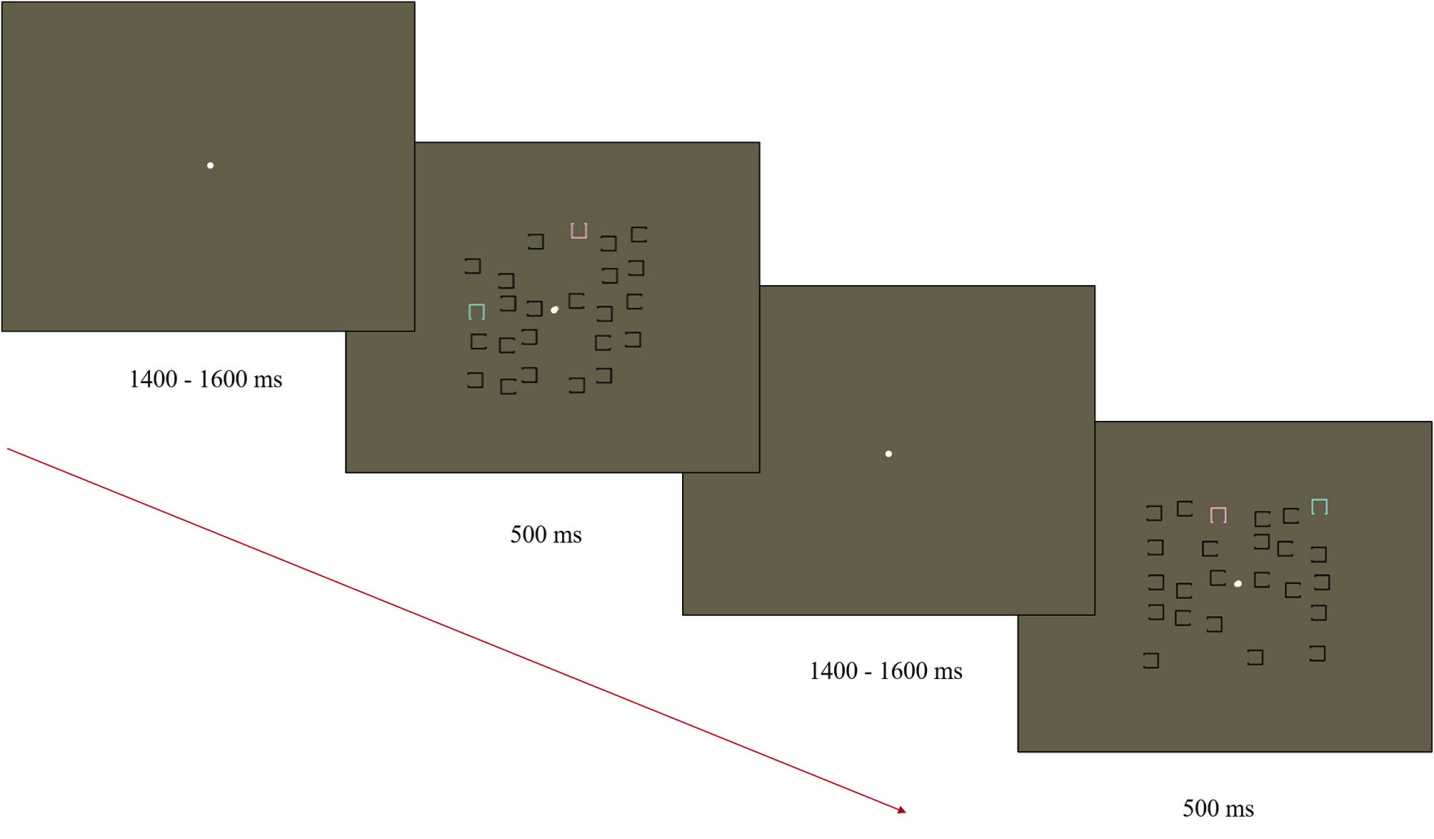
Diagram of the Visual Search Task used to elicit the N2pc. Participants were asked to find either the pink or blue “U” and indicate the direction of the open side (up/down).

Stimuli were presented on a 35.5 × 28 cm ViewPixx monitor (43.5 cm diagonal) using Presentation v 19.0 11.02.16 software (Neurobehavioral Systems). A Logitech Precision Game Pad recorded responses. A modified visual search task was used to elicit the N2pc based on the task used by Luck and colleagues (Luck et al., 2006; Kappenman et al., 2021; Figure 1). The stimulus display consisted of 12 objects in the left visual field and 12 in the right visual field. The objects in the display consisted of a square outline (0.45 × 0.45° visual angle) with a gap (0.3°) on one of the four sides. Objects were distributed within each half of the display randomly within an invisible box (2.5 × 5° visual angle). Objects were positioned starting 0.2° from fixation on either side of the display, with a minimum distance of 0.1° between items. Of the 12 objects in each visual field, 11 were black and had a gap either on the left or right side (randomly and independently determined). The remaining object was either blue (x = 0.28, y = 0.33, 116 cd/m^2^) or pink (x = 0.35, y = 0.30, 118 cd/m^2^) with a gap on the top or bottom (randomly and independently determined). Blue and pink objects were equidistant from the color of the gray background in CIE color space (1976). The locations of the pink and blue squares were randomized across trials and always presented in opposite visual fields to each other. Stimulus displays were presented for 500 ms with a central white fixation point (0.15° visual angle). The fixation remained visible during the jittered SOA of 1,400–1,600 ms (rectangular distribution average of 1,500 ms). The target object (blue or pink) was counterbalanced between participants with half of the participants asked to indicate the location of the gap for the pink squares in the first half of the experiment and blue in the second half. The order was reversed for the other half of participants. All responses were made using the index finger (upper gap trials) and middle finger (lower gap trials) of their dominant hand on a Logitech Precision Game Pad. Response mapping was maintained throughout the task and across participants as the mapping was natural (upper button press for gap on the top). In total, the task contained 230 trials, with a participant-mediated break provided every 40 trials.

#### Visual Working Memory Task (VWM)

Visual working memory was assessed via a delayed match-to-sample task of four colored objects (Luck and Vogel, 1997) from the BrainBaseline Cognitive Test Battery (https://www.brainbaseline.com/). The computerized assessment was administered on an IPad with a 9.7 in screen (Model MP2F2LL/A, IOS 10.3.2) with a display resolution of 2,048 × 1,536 pixels (264 ppi). This visual working memory assessment is based on Luck and Vogel's visual working memory task (Luck and Vogel, 1997), but unlike Luck and Vogel's task, the spatial locations of the encoded objects were not relevant for task performance. Participants were presented a display of four colored squares randomly selected out of 7 for 1,000 milliseconds [red RGB(1, 0, 0), green RGB(0,1, 0), blue RGB(0,0,1), yellow RGB(1,1, 0), purple RGB(0.88, 0.01, 0.89), black RGB(0,0, 0), cyan RGB(0.02,0.99, 0.78)]. Squares were 120 by 120 pixels and appeared at a center-to-center distance of 210 pixels. Squares were presented in a row above the fixation cross. Squares always appeared at the same four locations. Participants were asked to hold the squares in working memory during a 1,500 ms delay where a fixation appeared on the screen. They were then presented with a probe square, presented at the center of the screen below fixation, and asked to identify if the square matched one of the ones held in memory. Critically, the location of the colored squares did not need to be remembered, only the color of the squares. The task began with 10 practice trials and 68 test trials followed, 50% of which were “match” trials in which the probe square was part of the memory set.

#### Spatial Working Memory Task (SPWM)

Spatial working memory was assessed via a delayed match-to-sample task of four spatial locations (Awh and Jonides, 1998) from the BrainBaseline Cognitive Test Battery (https://www.brainbaseline.com/) on an IPad. Participants were presented with a central fixation point for 1,500 ms which was followed by a display with either 2 or 3 black dots (each 11 pixels radius) surrounding a central fixation for 500 msec. Locations were pseudo-randomly determined for each trial. Participants were asked to remember the locations of the dots. Following a 1,000 ms delay, a single red-dot probe was presented at one location on the screen and participants determined if the location of the red dot matched one of the dot locations from the memory array. Participants had 2,000 ms to respond while the probe dot remained on the screen and the trial was terminated either at 2,000 ms or upon their response. There were 8 practice trials and a total of 60 test trials, 50% of which were match trials.

It is important to note that both visual and spatial working memory involve holding visually presented information in memory for a brief duration (colored squares or locations) followed by a memory probe, but they differ in the involvement, or relevance, of spatial vs. visual information for task performance. For both working memory tests, the BrainBaseline test battery calculates an accuracy score which it recommends as the appropriate, validated measure of analysis (Lee et al., 2012).

### Electrophysiological Methods

Scalp electroencephalograms (EEGs) were recorded from 32 active Ag/AgCl electrodes (actiCAP, Brain Products GmbH, Gilching, Germany) mounted on an elastic cap and references to the average of the left and right mastoids (TP9/TP10) using the Brain Vision actiCHamp (actiCHamp, Brain Products GmbH, Gilching, Germany). Impedances were below 50 kΩ (typically below 25 kΩ) at the beginning of the experiment and kept below 50 kΩ throughout the experiment. Electrodes were placed at Fp1, Fp2, F3, Fz, F4, F7, F8, FC3, FC4, C3, Cz, C4, C5, C6, TP9, CPz, TP10, P3, Pz, P4, P7, P8, P03, P04, P07, P08, 01, Oz, O2 according to the international 10/10 system. The horizontal electrooculogram (HEOG) was recorded from electrodes placed lateral to the external canthi and the vertical electrooculogram (VEOG) was recorded from an electrode placed below the right eye (Fp2 was used in combination with this electrode to create a VEOG for analysis as a difference in voltage between upper and lower eye locations during offline pre-processing).

#### Data Analysis and Reduction

Data were exported into MATLAB and analyzed using the EEGLAB toolbox (Delorme et al., 2011; https://sccn.ucsd.edu/eeglab/index.php) and ERPLAB toolbox (http://www.erpinfo.org/erplab). First, EEGs were adjusted for DC offset by removing the mean value across the EEG and then filtered using a IIR Butterworth bandpass filter from 0.1 to 30 Hz (half amplitude cut off, 12 db/oct and 40 db/dec roll-off). Second, data were re-referenced off-line to linked mastoids (TP9/TP10). Channels that were consistently bad were replaced using interpolation based on all electrodes; no participants had consistently bad electrodes at the critical P07 or P08 electrodes. Of the initial 242 participants, five participant data sets contained one bad channel that was interpolated and of these, four data sets were excluded in the final analysis due to other exclusionary criteria. Continuous data were then segmented into epochs (200 ms pre-stimulus to 600 ms post-stimulus) for two conditions: Targets in the left visual field (TL) and Targets in the right visual field (TR). Only data from correct trials were included. Segments were baseline-corrected using the mean of the 200 ms pre-stimulus period. Artifacts in the data were addressed in two ways. First, trials were removed from analysis if they contained significant ocular artifacts (±100 μvolts at HEOG or VEOG) during stimulus presentation (±150 ms surrounding stimulus presentation). Second, ocular, muscle and other artifacts were identified and corrected for the entire trial length (200 ms pre-stimulus to 600 ms post-stimulus) using independent component analysis (ICA; RUNICA Makeig et al., 1997, Delorme and Makeig, 2004) and SASICA (Chaumon et al., 2015). While ICA was used to initially identify artifacts, SASICA was used for independent confirmation of artifact components to be removed. Following artifact identification via ICA and SASICA, components were visually inspected and any components that did not appear purely artifact in nature were “un-flagged” and kept in the data. For each participant's cleaned EEG data set, the trials for each condition were averaged. There was an average of 138.29 (SD = 18.16) trials per participant across all conditions with an average of 137.90 (SD = 18.47) for trials where the target was presented on the left and 138.69 (SD = 18.61) for when the target was presented on the right.

The N2pc was based on activity at the P07 and P08 electrodes and calculated as the difference between electrode sites contralateral and ipsilateral to the targets. To obtain accurate N2pc measures for our regression analysis, we selected the window for data analysis by collapsing across color (pink/blue) conditions and plotting the target-right and target-left waveforms. Although this approach determines the measurement window *post-hoc*, it does not provide any information about how the N2pc measures are related to the individual difference measures, and therefore does not bias our conclusions. Differences between left and right waveforms were seen within the 240–290 ms window, consistent with previous research (e.g., Gaspar et al., 2016). The validity of this window was confirmed using Mass Univariate Analysis by testing all electrodes as well as time-samples that could be expected to show an effect (100–400 ms post-stimulus onset) for significant differences (α = 0.05) using a permutation test over the tmax statistic to control for multiple comparisons (Groppe et al., 2011). Electrodes of interest here were P07/P08 and significant differences at these electrodes were seen between 206 and 336 ms post-stimulus presentation. Thus, the window used in analysis, 240–290 ms falls within this time-frame. In addition to amplitude, fractional area latency was calculated on the N2pc using a 50% fractional area within the 200–350 ms time window. Standard jackknife procedures (Miller et al., 1998) were used in analysis of fractional area latency data.

#### Data Analysis Approach

A one-way repeated measures ANOVA with a within-subject factor of Presentation Hemifield (2: contralateral, ipsilateral) was conducted on contralateral and ipsilateral amplitude data. Both F-value statistics and effect size as reflected in partial eta squared ($\eta_{\text{p}}^{2}$) are presented. This was done first to confirm the presence of the N2pc in amplitude data using IBM SPSS Statistics 25. Pearson correlations were calculated between all behavioral measures and N2pc measures. Additionally, all correlations were recalculated after excluding bivariate and univariate outliers. Univariate and bivariate outliers were identified using 1.5 interquartile range criteria (Aguinis et al., 2013). Table 1 provides descriptive measures and summarizes the correlations between working memory measures, visual search task performance, and N2pc measures; correlation values are presented with all data included as well as with outliers removed. Following this, multiple linear regression was used to examine the relation between visual search performance and N2pc amplitude using accuracy and response time as predictors of N2pc amplitude as the dependent variable. Multiple linear regression was also performed to examine the relation between working memory accuracy and N2pc amplitude using visual and spatial working memory accuracy to predict N2pc amplitude as the dependent variable. The same multiple linear regressions for N2pc fractional area latency data were performed using R i386 4.0.2. Thus, one multiple linear regression was conducted using visual search task accuracy and response time as predictors of N2pc fractional area latency as the dependent variable. A second multiple linear regression used visual and spatial working memory accuracy to predict N2pc fractional area latency as the dependent variable. All assumptions of multiple linear regression were met, including measures of collinearity measured by VIF, which were below 1.5 for both linear regressions.

**Table 1:** Means, standard deviations and correlations of behavioral and ERP measures.

	**Variable**	**Mean**	**Standard deviation**	**1**	**2**	**3**	**4**	**5**
1	N2pc amplitude (μV)	−1.64	0.98	X				
2	N2pc fractional area latency (ms)	277.10	16.44	0.283^**^ (0.277^**^)	X			
3	Visual search task accuracy (%)	87.47	9.19	0.243^**^ (0.242^**^)	0.002 (−0.011)	X		
4	Visual search task response time (ms)	524.32	59.17	0.132+ (0.131+)	0.264^**^ (0.299^**^)	0.253^**^ (0.216^**^)	X	
5	VWM accuracy (%)	84.7	7.29	−0.077 (-0.066)	−0.155^*^ (-0.116)	0.136 (0.056)	−0.174^*^ (−0.215^**^)	X
6	SPWM accuracy (%)	89.7	8.51	−0.183^**^ (−0.208^**^)	−0.061 (−0.035)	0.312^**^ (0.336^**^)	0.030 (0.001)	0.433^**^ (0.287^**^)

*+two-tailed trend p < 0.1*.

* *two-tailed significance p < 0.05*.

** *two-tailed significance p < 0.01*.

*() Correlations with bivariate and univariate outliers removed in parentheses*.

## Results

### Behavioral Analyses

Mean accuracy and response times for correct trials were calculated for each participant and condition of the visual search task. For the visual working memory (VWM) and spatial working memory (SPWM) assessments, proportion accuracy was calculated for each participant. For all three tasks, trials in which response times were <200 ms and >1,000 ms were excluded from analysis because they were attributed to anticipatory or inattention responses (>3 SD's from the grand mean), resulting in a loss of <2% of data.

**Figure 2:**
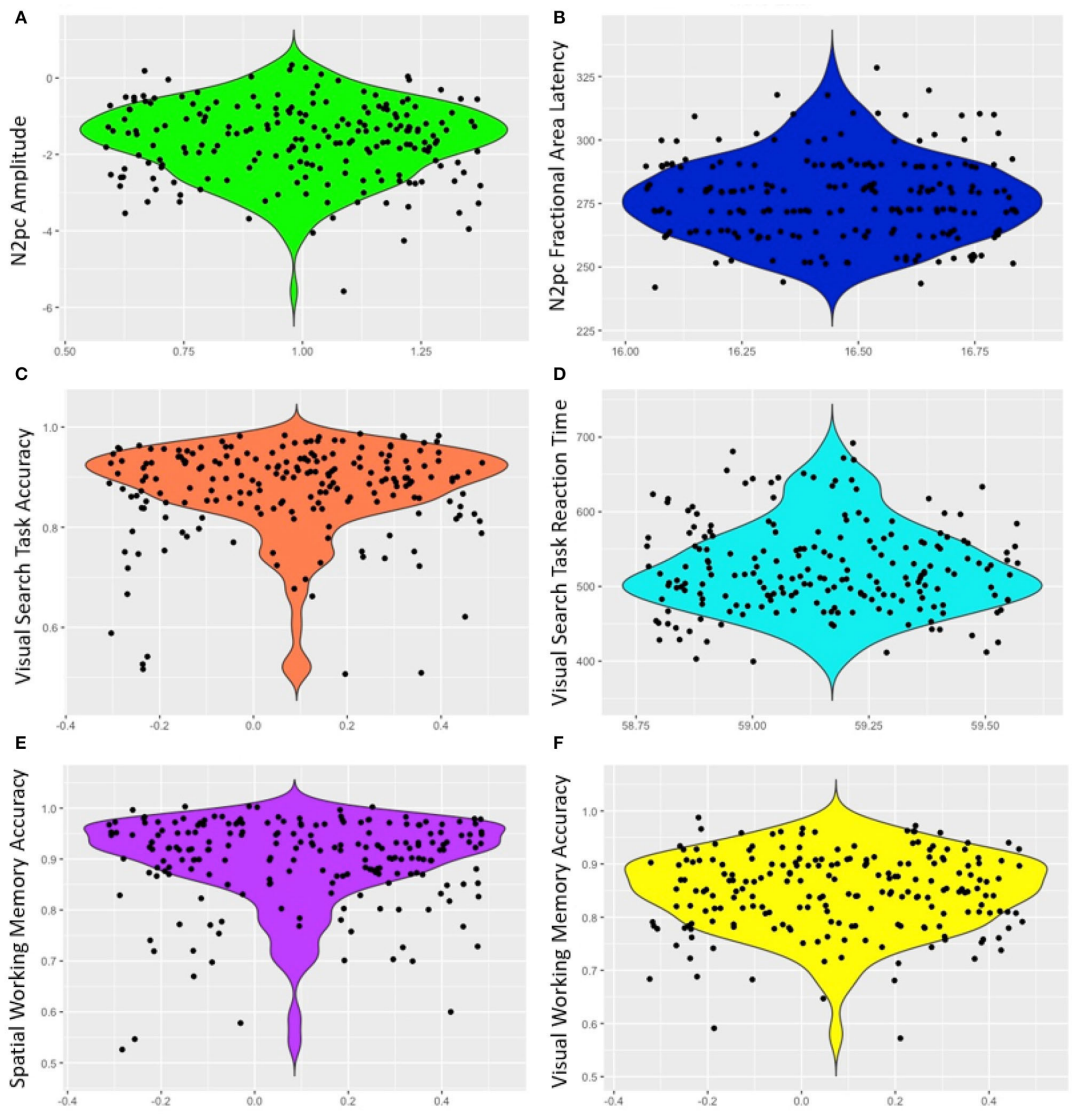
Violin plots of ERP and behavioral measures **(A)** N2pc amplitude, **(B)** N2pc fractional area latency, **(C)** Visual search task accuracy, **(D)** Visual search task response time, **(E)** Spatial working memory accuracy, and **(F)** Visual working memory accuracy. Points are jittered randomly for better visualization on the x-axis.

Mean response time for the visual search was 524.32 ms (*SD* = 59.17) and mean accuracy was 87.47% (*SD* = 9.18). The high level of accuracy on the task indicates that participants attended to the task (Table 1, Figure 2). Similarly, accuracy was high for both VWM (84.70%, *SD* = 7.29) and SPWM (89.70%, *SD* = 8.51); examination of the performance distribution suggests that the data contain enough variability for analyses despite possible ceiling effects (see Figure 2).

Correlation and multiple regression analyses were conducted to examine the relationship between working memory ability and visual search performance. SPWM scores were positively and significantly correlated with visual search task accuracy, indicating that those with higher scores on spatial working memory tended to have higher accuracy on the visual search task, but VWM was not correlated with performance accuracy. These results do not change when outliers are removed. The multiple linear regression model on visual search task accuracy with VWM and SPWM as predictors explained a significant proportion of the variance (*R*^2^ = 0.097, adj. *R*^2^ = 0.089, *F*_(2,202)_ = 10.909, *p* < 0.0001). SPWM had significant positive regression weights, indicating participants with greater spatial working memory ability are expected to be more accurate performing visual search, after controlling for the other variable in the model (*B* = 337; β = 0.312, *t*(202) = 4.203, *p* < 0.0001). VWM did not contribute to the model (*B* = 0.001, β = 0.001, *t* (202) = 0.015, *p* = 0.988). Although the two working memory measures are significantly correlated, a regression on just SPWM produced an identical β of 0.312, indicating that collinearity is not affecting the results. These results are consistent with other studies that show spatial working memory ability is related to visual search performance.

Response times for the visual search task were negatively correlated with VWM indicating greater VWM scores were related to faster RTs, but SPWM was not correlated with response times (Table 1). The multiple regression model on visual search response times with VWM and SPWM as predictors explained a significant proportion of the variance (*R*^2^ = 0.044, adj. *R*^2^ = 0.034, *F*_(2,202)_ = 4.62, *p* = 0.011). VWM had significant negative regression weights, indicating that those with greater VWM ability are expected to produce faster responses, after controlling for the other variable in the model (*B* = −186.341, β = −0.230, *t*(202) = 3.007, *p* = *0*.003). SPWM (*B* = 89.975, β = 0.129, *t*(202) = 1.695, *p* = 0.092) did not significantly contribute to the model.

### ERP Analyses

#### N2pc Amplitude

**Figure 3:**
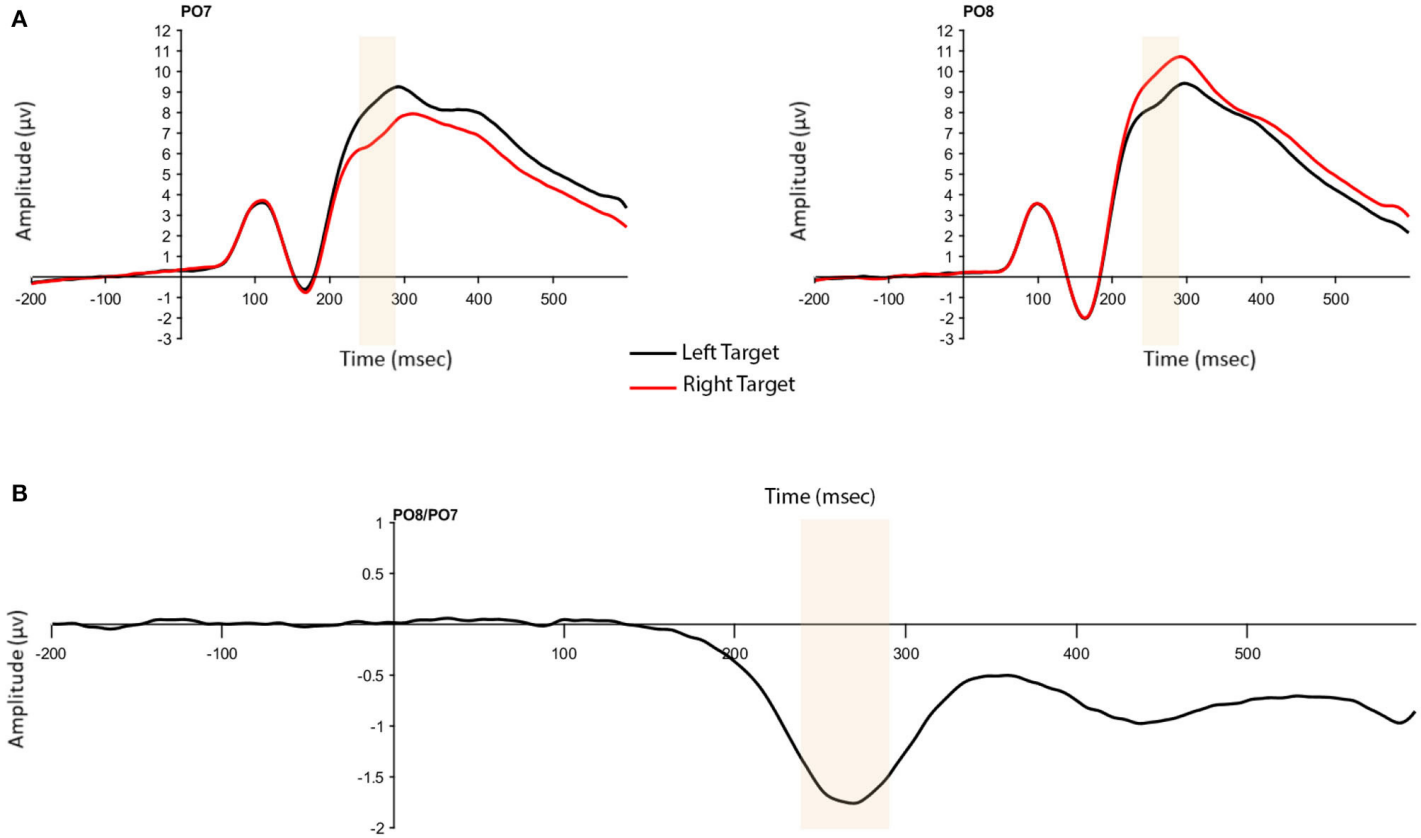
**(A)** N2pc to targets presented in the left and right hemifield at contralateral and ipsilateral electrodes (P07 and P08). **(B)** N2pc difference wave. Highlighted window is 240–290 ms in both figures.

The presence of the N2pc was confirmed by a significant one-way, repeated measures analysis of variance (ANOVA) with the within-subject factor of Presentation Hemifield (2: contralateral, ipsilateral). As expected, larger ERP amplitudes were found for voltages at contralateral compared to ipsilateral electrodes (*F*_(1,204)_ = 570.72, *p* < 0.001, $\eta_{\text{p}}^{2}$ = 0.737; Figure 3).

#### N2pc Amplitude and Visual Search Task Performance

Correlation and multiple regression analyeses were conducted to examine the relationship between N2pc amplitude and visual search performance (accuracy and correct RTs; Table 1). Visual search accuracy was significantly correlated to the N2pc amplitude (*r*(203) = 0.243, *p* < 0.001), but correct RTs only showed a trend relation to N2pc amplitude (*r*(203) = 0.132, *p* = 0.059). Moreover, removing bivariate and univariate outliers did not alter significance of these correlations (see Table 1). The multiple linear regression on N2pc amplitude with visual search accuracy and RTs as predictors explained a significant proportion of the variance (*R*^2^ = 0.099, adj. *R*^2^ = 0.090, *F*_(2,202)_ = 11.132, *p* < 0.001). Accuracy had significant negative regression weights, indicating participants with greater task accuracy had more negative N2pc amplitudes (*B* = −3.158, β = −0.296, *t*(202) = −4.283, *p* < 0.001) and RTs had significant positive regression weights, indicating that participants with longer responses produce less negative N2pc amplitudes (*B* = 0.003, β = 0.207, *t*(202) = 2.999, *p* = 0.003). These results confirm prior findings that greater accuracy and faster RTs on the visual search task are expected to produce larger N2pc deflections.

#### N2pc Amplitude and Individual Differences in Working Memory

**Figure 4:**
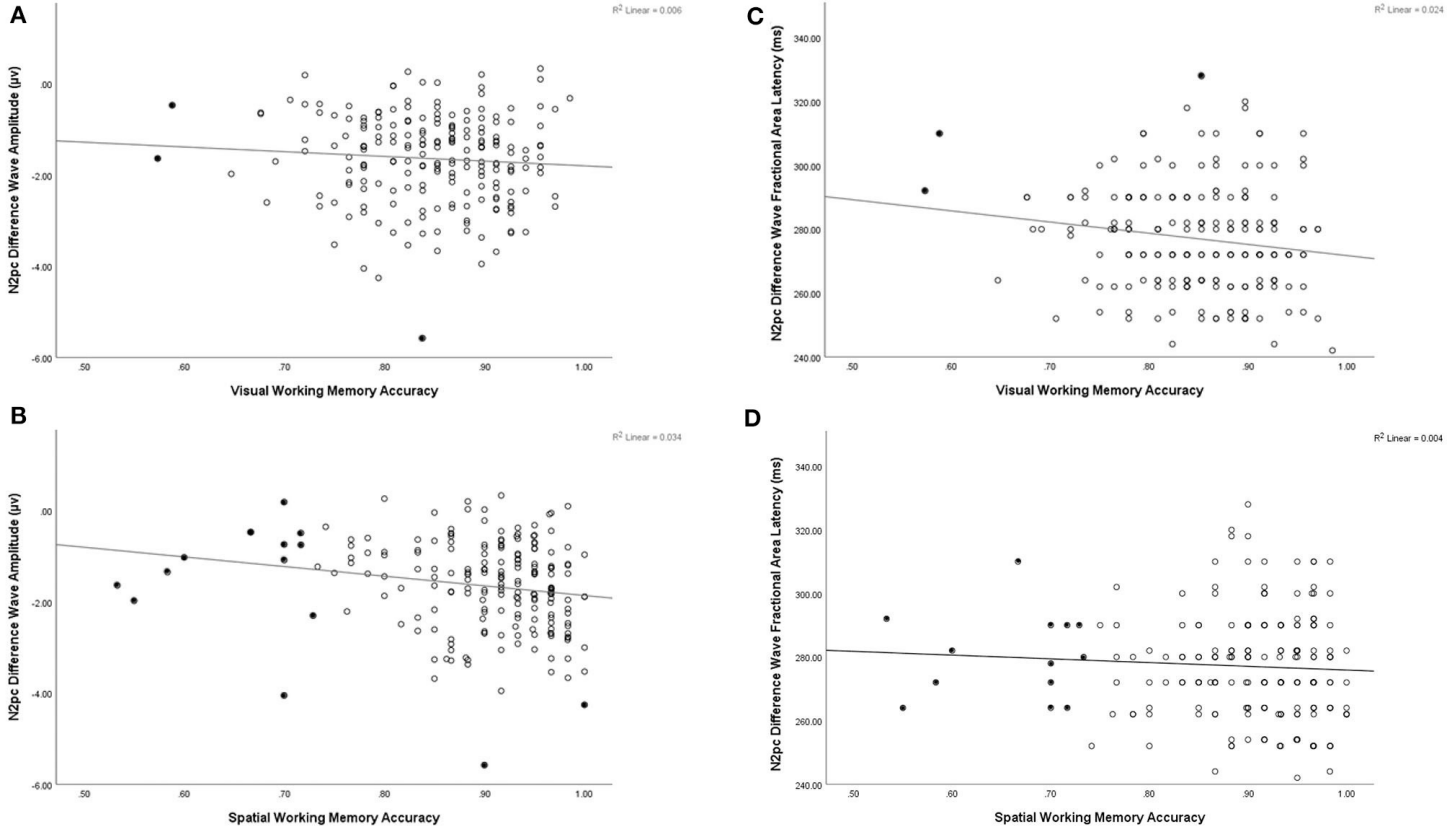
Illustrative scatter plots to show relation between N2pc and working memory tasks **(A)** N2pc amplitude plotted by accuracy on visual working memory task, **(B)** N2pc amplitude plotted by accuracy on spatial working memory task, **(C)** N2pc fractional area latency plotted by accuracy on visual working memory task, **(D)** N2pc fractional area latency plotted by accuracy on spatial working memory task. Filled in points represent data points excluded in correlations presented with bivariate and univariate outliers removed in Table 1.

Correlation and multiple regression analyeses were conducted to examine potentially differential contributions of VWM and SPWM abilities to N2pc amplitude (Table 1). SPWM showed a significant positive correlation with N2pc amplitude (*r*(203) = −0.183, *p* < 0.001), but VWM did not (*r*(203) = −0.077, *p* = 0.273). Similar to the previous analyses, removal of bivariate and univariate outliers did not alter the level of significance of the correlations. The multiple linear regression on N2pc amplitude with VWM and SPWM as predictors explained a significant proportion of the variance (*R*^2^ = 0.034, adj. *R*^2^ = 0.024, *F*_(2,202)_ = 3.513, *p* = 0.032). SPWM had significant negative regression weights (*B* = −2.130, β = −0.185, *t*(202) = −2.406, *p* = 0.017), suggesting that better spatial working memory is predictive of larger N2pc amplitudes (Figure 4). VWM did not significantly contribute to the model VWM (*B* = 0.040, β = 0.003, *t*(203) = 0.039, *p* = 0.969).

#### N2pc Latency and the Visual Search Performance

Correlation and multiple regression analyeses were conducted to examine the relationship between N2pc fractional area latency and visual search task performance (accuracy, response times). Significant correlations were found between N2pc latency and RTs (*r*(203) = 0.264, *p* < 0.001), but not accuracy (*r*(203) = 0.002, *p* = 0.997). Moreover, excluding bivariate and univariate outliers did not change the level of significance of these correlations. The multiple linear regression on N2pc latency with accuracy and response times as predictors explained a significant proportion of the variance (*R*^2^ = 0.074, adj. *R*^2^ = 0.065, *F*_(2,202)_ = 8.08, *p* < 0.001). Response times significantly contributed to the model and predicted latency (*B* = 0.078, β = 0.281, *t*(202) = 4.021, *p* < 0.0001), with the positive coefficient suggesting that longer response times predict longer N2pc latency. Accuracy did not significantly predict latency (*B* = −12.375, β = −0.069, *t*(202) = −0.988, *p* = 0.324).

#### N2pc Latency and Individual Differences in Working Memory

Correlation and multiple regression analyses were conducted to examine the relationship between VWM and SPWM and N2pc fractional area latency. A significant correlation was found between VWM and N2pc latency (*r*(203) = −0.155, *p* = 0.026), but not SPWM (*r*(203) = −0.061, *p* = 0.386). However, closer inspection of the data revealed that this correlation was driven by outliers and was therefore spurious: excluding univariate or bivariate outliers (three of the 205 participants) resulted in the loss of significance of the Visual Working Memory correlation with N2pc fractional area latency (*r*(200) = −0.116, *p* = 0.101). The same procedure did not alter the level of significance for SPWM. To further examine the relation between working memory and latency a linear regression was conducted using VWM and SPWM as the predictors of N2pc fractional area latency. The multiple linear regression on N2pc latency with VWM and SPWM as predictors did not reach significance (*R*^2^ = 0.024, adj. *R*^2^ = 0.015, *F*_(2,202)_ = 2.501, *p* = 0.085).

## Discussion

Previous research has suggested that working memory can affect the visual search process as well as the amplitude and latency of the N2pc. However, the pattern of findings across studies suggest there may be differential contributions of visual working memory and spatial working memory availability. Although some studies have manipulated working memory during visual search tasks to examine working memory contributions to visual search, few studies have examined working memory via individual differences in working memory abilities. In this study we investigated whether individual differences in visual and spatial working memory abilities differentially influence visual search processes as evidenced by task performance and the N2pc. We tested a large sample (*n* = 205) on visual working memory (VWM) and spatial working memory (SPWM) tasks independent of an EEG/ERP visual search task. Importantly, our results support and extend findings in the literature that suggest that *spatial working memory* in particular plays an important role in visual search performance and the N2pc (e.g., Oh and Kim, 2004; Woodman and Luck, 2004; Berggren and Eimer, 2018, 2019). SPWM ability significantly predicted visual search accuracy and N2pc amplitudes, such that greater SPWM ability led to greater task performance accuracy and greater negative N2pc amplitudes. Although greater VWM ability predicted faster responses in the visual search task, VWM did not significantly contribute to performance accuracy nor N2pc amplitudes. Neither SPWM nor VWM predicted N2pc latencies.

Thus, this study provides some interpretations and insights into equivocal findings in the literature regarding how working memory influences visual search processes and the N2pc. When both spatial and visual working memory abilities are assessed independently from the EEG/ERP visual search task, it is possible to confirm that pre-existing participant spatial working memory ability, more than visual working memory ability, can predict accurate visual search performance and N2pc amplitudes. Another benefit of measuring working memory ability outside of the visual search task is that it eliminates any confounds between working memory tasks with measurement of the N2pc (e.g., Störmer et al., 2013). Thus, spatial working memory ability relates to N2pc amplitudes beyond potential interference and/or availability while completing a task that requires both. Contrary to previous research, the data do not support a relation between SPWM or VWM and N2pc latency.

The present study found spatial working memory ability modulates N2pc amplitudes. However, visual working memory did not. Previous studies that have manipulated only visual working memory load and not spatial working memory load have not always found an N2pc effect. It may be that SPWM may contribute more to the N2pc elicited by visual search than VWM when the ability to select a target in a location is important. Specifically, spatial working memory ability may be important for visual search when spatial attention templates for target locations have to be held simultaneously in memory with multiple object features. It is not surprising to find that individual differences in SPWM influence the amplitude of the N2pc given that the N2pc is thought to index spatial attention. Spatial working memory, by definition, includes the use of spatial attention. Studies that use a spatial working memory task to elicit the N2pc support the finding that working memory processes interact with visual selective attention. For example, Störmer et al. (2013) found a moderate relation between SPWM capacity and the size of the N2pc effect in young adults (*r*(33) = 0.41).

It is important to note that even though we found reliable effects indicating that greater spatial working memory ability is correlated with more negative N2pc amplitudes, our effect size is small (*r*(203) = −0.183, slightly higher when outliers were removed r(189) = −0.208). Unlike some other studies, we did not measure the degree to which working memory contributed to visual search using concurrent tasks; rather, we measured individual ability in working memory based on performance on an independent task. Our question was whether, when measured by pre-existing ability, is spatial working memory or visual working ability predictive of visual search as indicated by N2pc amplitudes. We would therefore not expect the effect size in our study to be as large as those that manipulate working memory in concurrent/dual tasks. Further, one of the strengths of our study is that our large sample size allowed us to detect the relatively small effect of spatial working memory ability on visual search. No such relationship was indicated between visual working memory ability and N2pc amplitudes with the same sample. These results indicate that visual working memory and spatial working memory components contribute differently to the visual search task. When measured by pre-existing ability, spatial working memory is predictive and visual working memory is not. This finding is consistent with prior studies that visual search relies differentially on spatial vs. working memory (Oh and Kim, 2004; Woodman and Luck, 2004). Moreover, it is consistent with visual search task accuracy in this study which was significantly correlated with spatial working memory accuracy (r(203) = 0.312). However, another possibility for the smaller effect sizes seen in this study is that because the N2pc contains multiple subcomponents (e.g., Pd), each subcomponent may be influenced (or not) by spatial working memory in different ways as discussed further in later sections of this discussion. It is possible that future studies that are designed to isolate the different subcomponents of the N2pc and examine individual differences in SPWM, might show a larger effect size for one of these subcomponents if isolated from the N2pc component. Moreover, while this study was focused on working memory and the N2pc, the N2pc may be influenced by other cognitive processes not examined here (e.g., reorienting of attention, Galfano et al., 2011).

Although some studies find that visual working memory influences N2pc amplitudes, our data did not show such a relationship. One reason for this discrepancy is that to elicit the N2pc, it is necessary to present a lateralized stimulus, thus engaging spatial attention. Previous studies on visual working memory, despite using non-spatial items to be stored in visual working memory, nonetheless engaged spatial attention by presenting those stimuli laterally or asking participants to remember the items as a search array to obtain the N2pc (Kumar et al., 2009; Dell'Acqua et al., 2010; Berggren and Eimer, 2018, 2019). This additional engagement of spatial processing during the elicitation of the N2pc may account for previous findings.

One potential limitation of this study is that the N2pc may not fully reflect the contributions of working memory on its summed target and distractor suppression subcomponents, as is argued by Gaspar et al. (2016). They and others designed experiments to distinguish between two subcomponents in the full N2pc difference wave, one related to target processing and the other to distractor processing. Gaspar et al. (2016) found a significant correlation between the Pd subcomponent and visual working memory but not the target-related N2pc. Similarly, Henare et al. (2018) varied working memory load in a visual search task and found that it only affected the Ptc (similar to the Pd, Hickey et al., 2009) related to distractor suppression rather than target processing. If there are differences in subcomponent involvement for spatial working memory and visual working memory, it may be that spatial working memory is more highly correlated with the target processing subcomponent, and visual working memory is more correlated with the distractor processing subcomponent. To understand the neural processes involved in visual search, future experiments should not only assess both spatial and visual working memory, but also manipulate visual search difficulty (i.e., vary the number of distractors or target difficulty) to differentially affect target selection vs. distractor suppression processes.

In contrast to N2pc amplitudes, we found that neither visual nor spatial working memory abilities reliably predicted the N2pc latency. Although there initially appeared to be a significant correlation between visual working memory and N2pc latency despite a non-significant regression model, it failed to reach significance with the removal of three outlier data points (out of 205); similar outlier corrections for other measures did not change correlation significance. Thus, the present data do not strongly support claims of a relation between visual working memory and the latencies of the N2pc difference wave. Comparing our results to the literature, it is possible that studies that manipulate visual working memory load within the search task impact N2pc latency due to concurrent demands on spatial attention when eliciting the N2pc but when measured independently this effect is lost. In addition, the lack of a relation between working memory accuracy and N2pc latency may also arise from insufficient variability in the latency data. However, the latency data appears to have more spread than the amplitude data (Figure 2) suggesting that variability does not explain the lack of an effect.

Despite the large sample size, the current study has some limitations in terms of the restricted range of the working memory scores and the homogeneity of cognitive abilities of our participants. Although the visual working memory and spatial working memory measures that we used were derived from confirmed paradigms (Luck and Vogel, 1997; Awh and Jonides, 1998) and were administered via a validated test battery (https://brainbaseline.com; Lee et al., 2012), the measures did not provide sufficient high-level difficulty for our high-functioning, young-adult population to produce a large spread in the distribution of scores. At issue is that working memory measures that do not exceed working memory capacity such as those used in this study may be less reliable (Balaban et al., 2019). The fact that we did find consistent, reliable results showing the SPWM ability related to visual search accuracy and N2pc amplitudes and that it is consistent with other related studies attests to the strength of the finding. Unfortunately, although our results confirm some studies' lack of correspondence of VWM ability with visual search performance and N2pc measures, restricted range on the VWM may also have affected this aspect of our findings. To fully observe any differential contributions of different kinds of working memory capacity, future studies should address this issue by testing a participant population with a greater range of cognitive abilities using working memory assessments for a larger range of cognitive abilities.

In summary, spatial working memory ability appears to be important for visual search accuracy and N2pc amplitudes. Novel to this study, we used a large sample of participants to examine whether individual differences in both visual and spatial aspects of working memory ability relate to visual search processes indicated by the N2pc. When working memory ability was assessed independently of the experimental task, our findings indicated that spatial working memory - and not just working memory in general - aids visual search processes indicated by the amplitude of the N2pc. Specifically, the N2pc is larger for those with better spatial working memory. In contrast, when we assess visual working memory without a spatial component, we find that individual differences in visual working memory ability do not significantly influence amplitude. Finally, the present data do not support either visual or spatial working memory as predictors of N2pc latency. Clearly visual search requires both visual and spatial working memory to successfully detect a target among distractors, but our study further suggests that people's independent ability to maintain spatial information is more strongly related to behavioral and neural responses associated with selective attention in visual search than their ability to maintain visual information.

## Data Availability Statement

The datasets presented in this article are not readily available because the data set is part of an ongoing research project. It will be released and made available once all projects associated with the dataset are completed. Requests to access the datasets should be directed to jcouperu@mtholyoke.edu.

## Ethics Statement

The studies involving human participants were reviewed and approved by Hampshire College Institutional Review Board, Claremont McKenna College Institutional Review Board, and University of Richmond Institutional Review Board. The participants provided their written informed consent to participate in this study.

## Author Contributions

JC: conceptualization, project administration, funding acquisition, resources, supervision, methodology, investigation, formal analysis, and roles/writing – original draft, review, and editing. KL and JH: conceptualization, investigation, data curation, and software. JR: conceptualization, investigation, and data curation. AL: conceptualization, investigation, and data curation. CB and CR: conceptualization, funding acquisition, resources, investigation, data curation methodology, and writing – review and editing. All authors contributed to the article and approved the submitted version.

## Conflict of Interest

The authors declare that the research was conducted in the absence of any commercial or financial relationships that could be construed as a potential conflict of interest.
